# Genetic analysis of a bronze age individual from Ulug-depe (Turkmenistan)

**DOI:** 10.3389/fgene.2022.884612

**Published:** 2022-08-22

**Authors:** Perle Guarino-Vignon, Nina Marchi, Amélie Chimènes, Aurore Monnereau, Sonja Kroll, Marjan Mashkour, Johanna Lhuillier, Julio Bendezu-Sarmiento, Evelyne Heyer, Céline Bon

**Affiliations:** ^1^ Eco-Anthropologie (EA), Muséum National D'Histoire Naturelle, CNRS, Université de Paris, Paris, France; ^2^ CAGT, UMR 5288, CNRS, Université Paul Sabatier Toulouse III, Toulouse, France; ^3^ CMPG, Institute of Ecology and Evolution, University of Berne, Berne, Switzerland; ^4^ BioArCh, Department of Archaeology, University of York, York, United Kingdom; ^5^ Archéozoologie, Archéobotanique Sociétés, Pratiques et Environnements (AASPE), Muséum National D'Histoire Naturelle, CNRS, Paris, France; ^6^ Archéorient, Environnements et Sociétés de L'Orient Ancien, CNRS/Université Lyon 2, Lyon, France

**Keywords:** Paleogenetics, Central Asia, Bactrian-Margian Archaeological Complex, Bronze Age, Turkmenistan

## Abstract

The Oxus Civilisation (or Bactrio-Margian Archaeological Complex, BMAC) was the main archaeological culture of the Bronze Age in southern Central Asia. Paleogenetic analyses were previously conducted mainly on samples from the eastern part of BMAC. The population associated with BMAC descends from local Chalcolithic populations, with some outliers of steppe or South-Asian descent. Here, we present new genome-wide data for one individual from Ulug-depe (Turkmenistan), one of the main BMAC sites, located at the southwestern edge of the BMAC. We demonstrate that this individual genetically belongs to the BMAC cluster. Using this genome, we confirm that modern Indo-Iranian–speaking populations from Central Asia derive their ancestry from BMAC populations, with additional gene flow from the western and the Altai steppes in higher proportions among the Tajiks than the Yagnobi ethnic group.

## Introduction

Central Asia was one of the first regions outside of Africa populated by *Homo sapiens*. It has played a key role in human history, having served for millennia as a connection between Europe and Asia on the one hand, and Siberia and southern Eurasia on the other. This area exhibits a high genetic, linguistic, and ethnological diversity. Today, several ethnic groups are present in Central Asia belonging to two linguistic families: Indo-Iranian—composed of the Tajik and Yagnobi populations, traditionally sedentary agriculturalists—and Turco Mongol that encompasses Uzbeks, Turkmens, Karakalpaks, Kazakhs, and Kyrgyz, traditionally nomadic herders and organized into patrilineal descent groups.

Genetic analyses led by our team on about 40 Central Asian populations brought to light a major impact of social organization and cultural practices on Central Asia’s genetic diversity. The patrilocal rule of residence favors women’s migrations while increasing the spatial, linguistic, and ethnic structuring of men (Heyer et al., 2009; Marchi et al., 2017) and could explain the larger genetic differentiation between populations for the paternally inherited Y chromosome than for their maternal equivalent, the mitochondrial DNA. The male effective population size, but not the female one, is found to be smaller for the patrilineal Turco-Mongols than for the cognatic Indo-Iranian populations (Ségurel et al., 2008), suggesting an effect of the filiation rule. Indeed, the patrilineal filiation rule drastically decreases their masculine effective population size (Chaix et al., 2007; Marchi et al., 2017; Ségurel et al., 2008) and accentuates the male transmission of reproductive success Heyer et al., (2015). Eventually, by contrasting estimated genetic and given historical ages for ethnic groups and tribes, we unveiled that ethnicity and tribalism in Central Asia are likely cultural constructions rather than biological entities (Quintana-Murci et al., 2004; Heyer et al., 2009; Marchi et al., 2017). These cultural behaviors are expected to affect the demographic history of Central Asian groups (Aimé et al., 2013; Aimé, Heyer, and Austerlitz 2015; Balaresque et al., 2015).

Genetic analyses have also helped to understand the origin and history of these populations. First, they evidenced that the two cultural groups are genetically distinct (Martínez-Cruz et al., 2011; Heyer et al., 2009; Marchi et al., 2018, 2017). Indo-Iranian speakers have the greatest proximity to modern Western Eurasian populations, while the Turco-Mongols are mostly related to Eastern Eurasians (Northern Asia). However, the Turkmens stand out from this general conclusion: despite speaking a Turco-Mongol language, they are more related to Indo-Iranian populations, suggesting a recent change, both in their language and way of living (Guarino-Vignon et al., 2022). Second, using approximate Bayesian computation, our group inferred that the Indo-Iranian group resulted from the first prehistoric admixture between Western and Eastern Eurasian groups (Palstra, Heyer, and Austerlitz 2015). Then, some 2.3 ky ago, the Turco-Mongol group emerged from a second admixture between these proto–Indo-Iranians and Eastern Eurasians. However, genetic analyses based only on modern data can be skewed by the recent and population-specific demographic history of the populations taken as references.

The development of paleogenomics over the last 15 years has allowed a better understanding of the demographic events that shaped the history of these populations. In this region with a complex migration history, ancient DNA is precious for disentangling the different waves of migration. Furthermore, relying on precise archaeological context made possible the joint study of cultural and demographic changes through time.

Notably, the Neolithic way of life developed in the steppe territory in the North (corresponding to present-day Kazakhstan, Kyrgyzstan, and Northern Uzbekistan) around 4000 BC with the Botaï culture (de Barros Damgaard et al., 2018). Later, during the Middle Bronze Age (around 2000 BC), the Botaï culture was replaced by people associated with the Sintashta culture, responsible for the introduction of wheeled chariots and horse breeding in the steppes. Sintashta being related both from a cultural and genetic point of view to Western steppe groups, their presence in Northern Central Asia during the Bronze and Iron Age (third-first mill. BC) suggests some West-to-East migrations (Allentoft et al., 2015; Jeong et al., 2019), as well as great mobility linked to pastoralism (Bendezu Sarmiento, 2007; Bendezu Sarmiento, 2021a). During the Middle-Late Bronze Age, there seems to be a continuity with the Andronovo complex that was derived from the Sintashta horizon. At the end of the Bronze Age—the beginning of the Iron Age, some East Asian populations expanded in this area, reflecting the likely onset of Turco-Mongol expansion westwards (Hollard et al., 2014; Unterländer et al., 2017).

In the South (present-day southern Uzbekistan, Tajikistan, Turkmenistan, southern Kyrgyzstan, northern Afghanistan, and Northeastern Iran), agropastoral communities are present since 6000 BC (D. R. Harris, Gosden, and Charles 1996) and as early as the eighth millennium BC in Northeastern Iran (Harris 2011; Roustaei, Mashkour, and Tengberg 2015; David R). These groups are genetically similar to Neolithic Iranian communities (Broushaki et al., 2016; Lazaridis et al., 2016), which may suggest that the agricultural way of life was acquired through the expansion of Southwestern Eurasian farmer populations in the south of Central Asia or that the local hunter-gatherer ancestry was related to a vast population found in Iran and Caucasus (Shinde et al., 2019). Through the Chalcolithic and the Bronze Age, the development of agriculture is associated with increasing size of the villages and the beginning of irrigation, which culminated with the blossoming of the Bactrian-Margian Archaeological Complex (BMAC) also called the Oxus Civilization (Lyonnet and Dubova, 2020). Genetic data obtained from several archaeological sites show a strong genetic continuity between the Neolithic and the beginning of BMAC, with only a limited genetic contribution of other groups. BMAC displays the first structured proto-urban cities of the area gathering thousands of individuals, and a deep social structuring (Muradov, 2021). BMAC was part of the “Middle Asian Interaction Sphere,” a dynamic network of cultural interaction and interregional exchanges with the Indus Civilisation (northern India and Pakistan), the Syro-Anatolian area, Mesopotamia, and the Iranian Plateau (Possehl, 2002; Mutin et al., 2017). During the BMAC period, and more frequently after the Middle Bronze Age, some outliers, coming from Southern Asian or steppe populations are evidenced, suggesting that the long-distance relationships seen in the material assemblage reflect on the genetic level. For still unknown reasons, the Late Bronze Age (ca. 1800–1500 BC) corresponds to a major cultural, economic, and ideological shift in southern Central Asia, leading to the disappearance of the Oxus Civilisation and is characterized by deterioration in the quality of the craft industry and the disappearance of long-distance exchanges within the Middle Asia Interaction Sphere. However, contacts with neighboring steppe Andronovo cultural community increased during the Late and Final Bronze Age period (Rouse and Cerasetti, 2014). The following Iron Age is characterized by a mosaic of cultures, characterized by specific handmade pottery, with red geometric designs, also known as “Yaz I cultures,” which spread over a territory larger than the BMAC territory with a radical transformation of the settlement pattern, small sedentary villages replacing large proto-urban sites and spreading to new areas (Lhuillier, 2013; Lhuillier 2019). Because funerary practices change at this time, and inhumation is replaced by the exposition of corpses and defleshing by scavengers (Bendezu Sarmiento and Lhuillier 2013), the number of human remains for this period is low. For instance, genome-wide data have been published only for one individual from Turkmenistan Damgaard et al., (2018), whose ancestry is the result of an admixture between BMAC population and a steppe population related to Andronovo culture. Comparison of this individual with modern Indo-Iranians suggests genetic continuity since the Iron Age, as 90% of Yagnobis ancestry is inherited from BMAC, with only a limited pulse from an East Asian population and, for the Tajik group, from South Asia (Guarino-Vignon et al., 2022).

**FIGURE 1:**
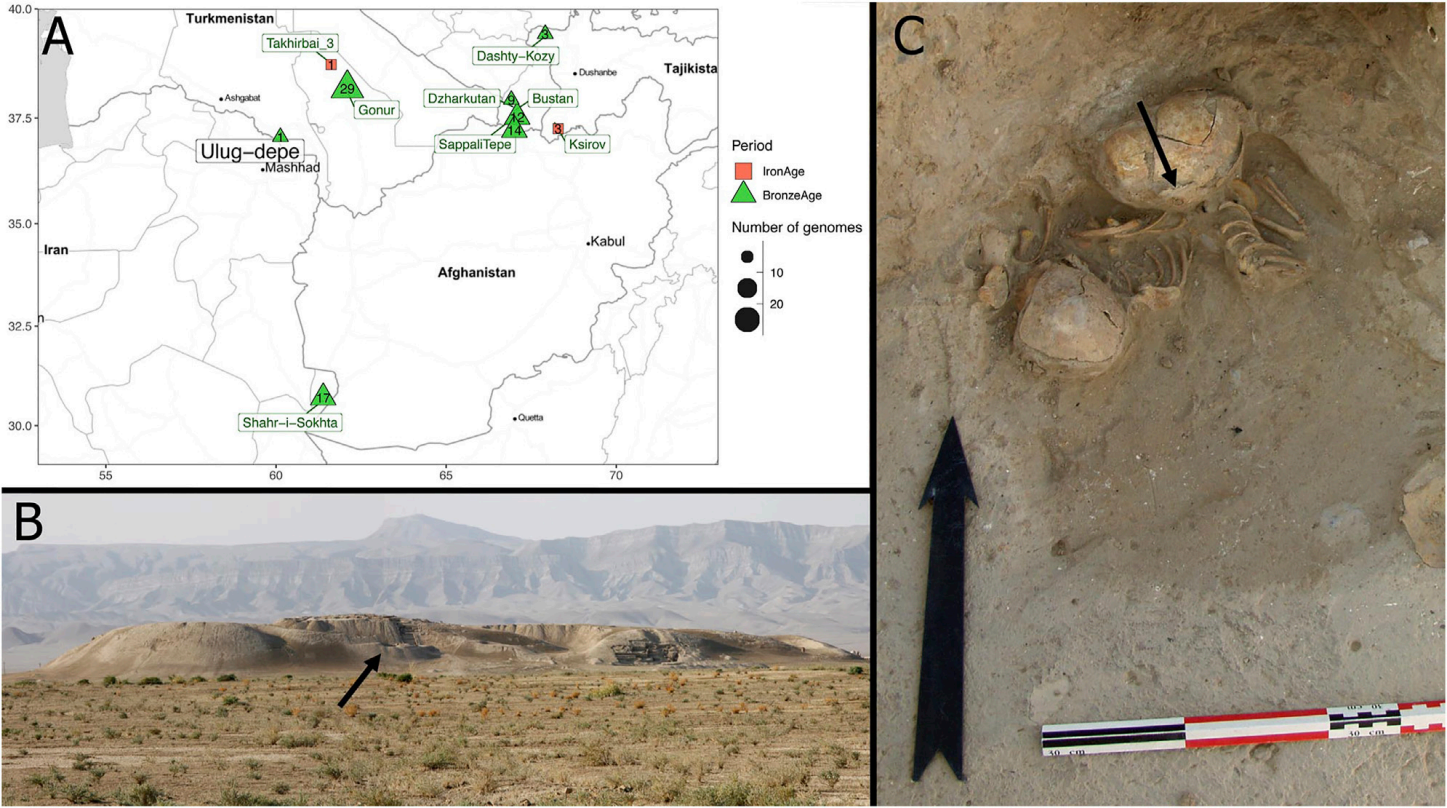
The archaeological context of *ULG75*. **(A)** Maps showing the different Turan Bronze Age (green) and Iron Age (red) sites from which genome-wide data were obtained. **(B)** View from Ulug-depe site from North-East. The place where *ULG75* was found is indicated by an arrow. **(C)** Photograph of the *ULG75* burial (grave 75). An arrow indicates the sample from which DNA was extracted.

Despite the publication of genome-wide data from several BMAC archaeological sites, none has been published up to now from Ulug-depe (Turkmenistan). Ulug-depe is one of the biggest proto-urban sites of the BMAC (13 ha), and is located in the formative area of the BMAC, halfway between Namazga-depe and Altyn-depe (Figures 1A,B), at 175 km east of Ashgabat in Turkmenistan. The site was first studied by V.I. Sarianidi in the 1960s and by the MAFTur in the early 2000s. Ulug-depe displays the longest stratigraphical sequence of southern Central Asia, from Early Chalcolithic to the Middle Iron Age, making it a key site for the understanding of the genesis and the evolution of the BMAC.

No necropolis has been (yet) found in Ulug-depe, but up to 100 burials from the Bronze Age have been discovered inside the houses (Bendezu Sarmiento, 2021b). Inside a house in trench 1 Est (Figure 1C), a grave was installed in the corner of a room that was likely still occupied. The pit grave contained the remains of three perinatal juveniles (Julio, 2013; Bendezu Sarmiento, 2021b).

To better understand the genetic diversity of Bronze Age Ulug-depe, we performed a paleogenetic analysis of one of these individuals.

## Materials and methods

### Material

Thirteen samples from the BMAC period and three from Iron Age have been selected, based on the overall preservation (Supplementary Table S8). They span several occupancies of the site from the Early Bronze Age (Namazga IV) to the Iron Age. The selected samples came from different areas of the skeleton: teeth, phalanx, and coastal fragments, as well as a petrous bone for one individual (grave 75). It belongs to an infant, around 10 months old, from the Middle Bronze Age (Namazga V period). The child was lying on the left side, in a crouched position, the head facing west. The bone deposit suggests that some disturbances have taken place, maybe when the last infant was buried.

### Ancient DNA extraction

All the preamplification steps were carried out in the clean room, dedicated to ancient DNA, of the Paleogenomic and Molecular genetics platform, set in the Musée de l’Homme (Paris, France). Ancient DNA extraction was performed for the 15 samples using a protocol adapted from Dabney et al. (2013). Briefly, 50–100 mg of the petrous bone were powdered by drilling and incubated in 1 ml of lysis buffer (0.45 M EDTA, 10 mM Tris-HCl (pH 8.0), 0.1% SDS, 65 mM DTT, and 0.5 mg/ml proteinase K) at 37°C for 14 h. After centrifugation, 1 ml of supernatant was recovered and purified with 13 ml of binding buffer (5M GuHCl, 40% 2-propanol 0.05% Tween 20, 90 mM sodium acetate 2M, 1× phenol red). The mixture was then transferred on a High Pure Extender Assembly column (Roche High Pure Viral Nucleic Acid Large Volume Kit) and centrifuged. Then, the column was washed following manufacturer recommendations (briefly, 500 µl of inhibitor removal buffer, centrifugation, and 450 µl of wash buffer twice). DNA was eluted in 100 µl of elution buffer. The amount of human DNA in the samples was evaluated through PCR amplification. PCR was performed in a 12-µl reaction volume containing mock or ancient DNA extracts, 300 pM sense (TGG​GGA​AGC​AGA​TTT​GGG​T) and antisense (TGG​CTG​GCA​GTA​ATG​TAC​G) primers targeting the mitochondrial DNA, 200 µM dNTP, 2.5 mM MgCl_2_, 1 × PCR buffer II, and 1 U of AmpliTaq Gold DNA polymerase (Applied Biosystems). 1 µl of PCR product was loaded on a 2% agarose gel to check for positive results.

### Library preparation

The ancient DNA extract was converted into a TruSeq Nano Illumina library following the manufacturer’s protocol with slight modifications that account for the ancient DNA damage. First, DNA was not fragmented; only 25 µl of ancient DNA extract was used; after end-repair, the libraries were purified using a MinElute column (Qiagen ^©^); libraries were amplified using 10–12 PCR cycles and purified on a MinElute column (Qiagen ^©^). Analyses on a LabChip ^®^ GX provided an estimated size distribution of fragments with a peak length of 150–250 bp.

Genome-wide data was successfully produced from the only sample (*ULG75* from grave 75) with more than 1% of endogenous DNA content. For this sample, a genomic capture was performed using the myBaits Expert Whole Genome Enrichment (WGE) kit (Arbor Biosciences) and the manufacturer’s instructions were followed. Baits were formed from the genomic DNA of three individuals of different (African, European, and Asian) ancestries. After enrichment, libraries were re-amplified using 12 PCR cycles.

### Sequencing

Captured libraries were sequenced on a NextSeq 500 (2 × 75 bp) instrument on the IGenSeq platform.

### Data processing

We similarly processed the new sample as follows. We mapped sequencing reads to the human reference genome (GRCh37) using BWA-0.1.17 (Li and Durbin, 2009) with the *aln* command and the parameter “−l 1,024.” Mapped reads were filtered out for mapping quality <25 with SAMtools 1.940. Duplicates were removed using Picard MarkDuplicates (http://picard. sourceforge.net), and the reads were realigned using GATK 3.5 IndelRealigner (Auwera et al., 2013). Finally, MapDamage 2 (Jónsson et al., 2013) was used to rescale base quality for all samples and take into account ancient DNA–specific damages. We restricted our analysis to known present-day DNA variants to minimize false positives. We used the *mpileup* command of SAMtools 1.9 (Li et al., 2009) to extract reads overlapping known variants from the v42.4 available at https://reich.hms.harvard.edu/allen-ancient-dna-resource-aadr-downloadable-genotypes-present-day-and-ancient-dna-data covering 1,233,013 positions SNPs (1240k dataset). For positions with more than one base call, one allele was randomly chosen with a probability equal to the frequency of the base at that position.

We calculated the contamination rate on the genome using AuthentiCT (Peyrégne and Peter 2020).

Biological sex was determined using Rx (Mittnik et al., 2016) and Ry (Skoglund et al., 2013) statistics. Mitochondrial haplogroup was found using Haplogrep v2.4.0 (Kloss-Brandstätter et al., 2011) and compared to the AmtDB database (Ehler et al., 2019).

### Merging genomic data

Among ancient human genomes from Eurasia from Paleolithic to Middle Age (Supplementary Table S1), DNA sequences were generated with whole genome shotgun or hybridization capture technics, from the 1240k dataset, and from Skourtanioti et al. (2020) and Jeong et al. (2020), we retained 1,587 ancient unrelated Eurasian individuals with more than 10,000 SNP hits on the 1240 k panel from the Human Origin dataset. We merged our individual with these 1,587 published individuals using *mergeit* from eigensoft. This first merge is called the “1240 k-dataset” and includes 1,233,013 SNPs. We provide all metadata about the ancient dataset in Supplementary Table S1.

For some analyses, it was then merged with 22 modern Indo-Iranian genomes [as described in Guarino-Vignon et al. (2022)] and included 716,743 SNPs.

For analysis requiring more modern diversity, we selected 3,109 published modern genomes from Eurasia, Mbuti population (the latter to serve as outgroup), from the Human Origin dataset and we haploidized them by randomly selecting one allele per position. We merged the modern dataset with the 1240 k-dataset as before, in order to provide a frame of comparison regarding the modern Eurasian diversity. The final merge was called “HO-dataset” and included 597,573 SNPs for 4,696 individuals.

### Descriptive analysis

We ran principal component analyses (PCA) with *smartpca* (Patterson, Price, and Reich 2006) on the HO-dataset for 1,309 European and Middle-Eastern modern individuals, and we projected all the ancient samples. We used the default parameters with *lsqproject: YES*, and *numoutlieriter: 0* settings.

We computed ADMIXTURE analysis (Alexander, Novembre, and Lange 2009) on the HO dataset, downsampling all populations to a maximum of 20 individuals, that is, for 1,266 Eurasian modern individuals, including East Asians, and 2,526 ancient samples. A subset of 365,075 SNPs were retained for the analysis after pruning for linkage disequilibrium done by using *plink 1.9* --indep-pairwise 200 25 0.4 function (Chang et al., 2015). We ran 10 replicated ADMIXTURE analyses for K between 2 and 15, from which we kept the most likely.

### D-statistics and f3 statistics

We performed D-statistics on the “1,240 k dataset” using the *qpDstat* program of the ADMIXTOOLS package (Patterson et al., 2012). We computed D-statistics of the form D(Mbuti, Y; BMAC populations, *ULG75*), with *Y* being ancient Paleolithic, Mesolithic, or Neolithic populations from Western Eurasia and most particularly southern Central Asia to test the proximity of the Ulug individual *ULG75* with other BMAC and post-BMAC groups. We also computed all D (Mbuti, Y; Indo-Iranian, *ULG75*), with Y being protohistoric Eurasian populations, and Indo-Iranians being Tajiks and Yagnobis. We corrected the Z-score of every D-statistic accounting for the repetitive testing using Benjamini and Hochberg’s (1995) method. We used *qp3pop* of the same package, using *inbreed: YES* parameter to compute f3-outgroup statistics f3 (Mbuti, BMAC population, ancient Eurasian population) to identify the Eurasian populations that share the highest genetic drift with Ulug-depe and four other BMAC groups. We only retained f3-statistics calculated on more than 50,000 SNPs.

### qpAdm analysis

We performed rotating *qpAdm* analysis from ADMIXTOOLS package, using the “1,240 k dataset,” to model the ancestry of *ULG75*, with the same set of populations as in Narasimhan et al. (2019).

We also performed the cladality test of *ULG75* with other BMAC populations compared to outlier populations from the region (Indus periphery pool, Shahr I Sokhta, and Seh Gabi) using qpAdm, with only BMAC as a source and the outliers added to the outgroups.

### Uniparental markers

Y chromosome and mitochondrial haplogroups were determined by comparison of *vcf* files to databases: respectively, Phylotree 17 (Oven and Kayser, 2009) using the software Haplogrep v2.241 (Kloss-Brandstätter et al., 2011); and ISOGG version 11 July 2020.

## Results

For 15 samples, no or weak PCR signal was observed, indicating that ancient DNA was not well preserved in these samples. PCR amplifications were robustly possible only for *ULG75*. We generated genome-wide data for this individual dated to the Bronze Age from the site of Ulug-depe in Turkmenistan, associated with BMAC. We obtained damage profile compatibles with ancient DNA (Supplementary Figure S1), and the estimation of contamination was low (0.047). The estimation of Rx (1.09+/−0.06) and Ry (0.0159+/−2 E-7) statistics shows that *ULG75* is a female. We obtained 133,761 SNPs, which is sufficient for genome-wide analyses.

### Mitochondrial DNA analyses

Mitochondrial haplogroup of *ULG75* is HV + 3,197 + 12,358 +16,311. The HV haplogroup is widely present in western Eurasia since the Neolithic, but this haplotype is rare. It has currently not been evidenced in any ancient DNA database, but the HV + 16,311 haplotype is present in two Central European Bell Beaker individuals (Ehler et al., 2019).

### Genetic affinity of the Ulug individual with other BMAC individuals

**FIGURE 2:**
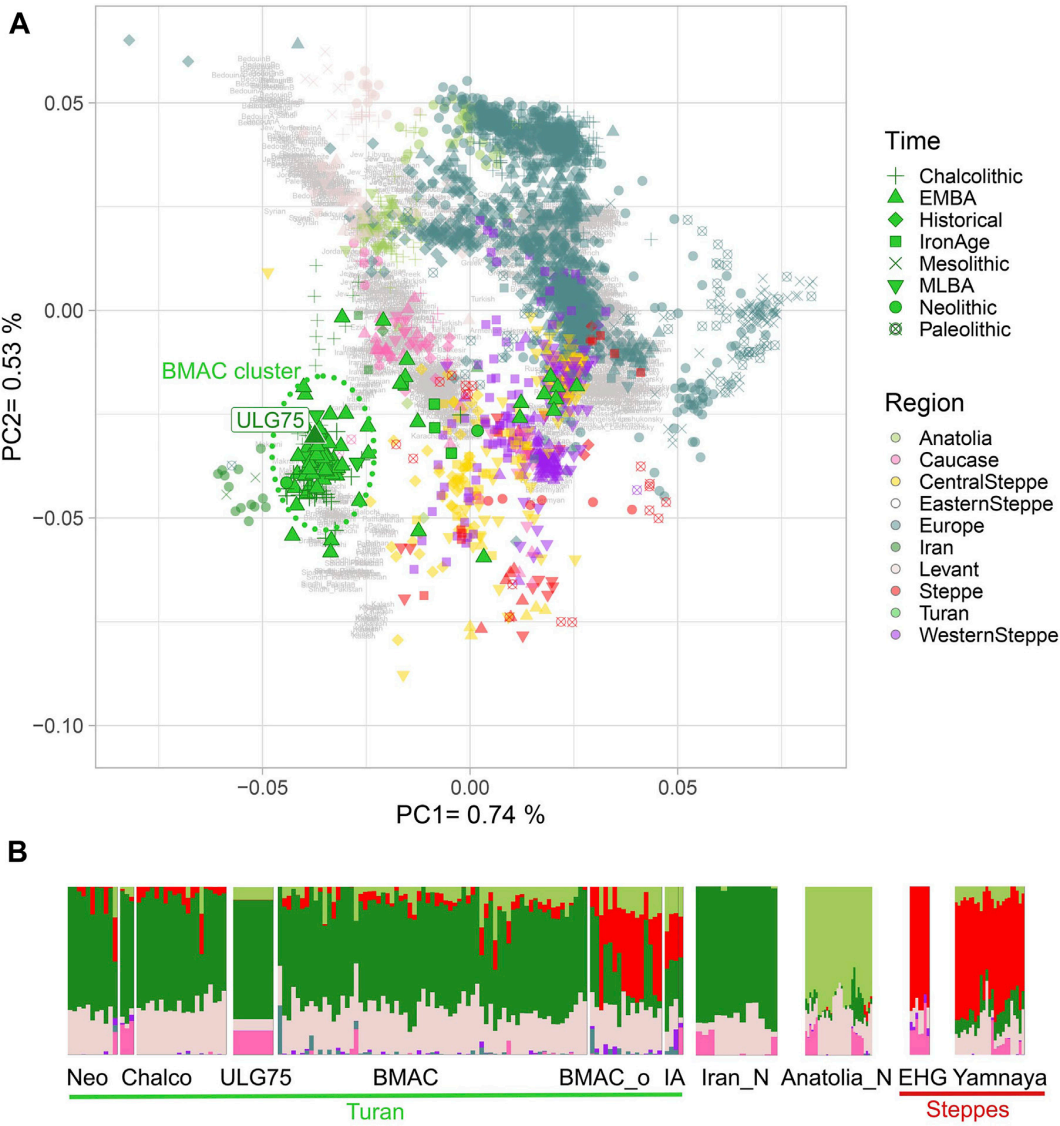
Genetic affinity of *ULG75* with other Turan individuals. **(A)** PCA is calculated on present-day individuals with ancient individuals projected onto it. Ancient Turan individuals are shown in non-transparent green with outlines. *ULG75* individual is labeled. **(B)** Unsupervised ADMIXTURE analysis was performed on the HO-dataset. Only *ULG75*, ancient Turan individuals, Neolithic Iranian Anatolian farmers, and Paleolithic and Eneolithic steppe populations are shown. Full ADMIXTURE is available in the supplementary data. Neo, Neolithic; Chalco, Chalcolithic; BMAC_o, outliers from BMAC sites; IA, Iron Age; Iran_N, Neolithic Iranian farmers; Anatolia_N, Neolithic Anatolian farmers; EHG, Eastern hunter-gatherers.

To decipher the genetic relations between the new Ulug-depe individual and other ancient populations belonging to BMAC, we calculated a PCA on the modern individuals of the “HO-dataset” and projected ancient individuals (Supplementary Table S1): *ULG75* falls within all other individuals of the Oxus civilization (Figure 2), including those from Dzharkutan or Gonur-Depe. To further explore the genetic similarity of our Ulug sample with the other individuals from BMAC, we performed an ADMIXTURE analysis (Alexander, Novembre, and Lange 2009). Previous analyses of BMAC individuals have shown that these sites receive individuals from other origins (Steppe populations and South-Asian populations), later identified as outliers. *ULG75* presents a profile close to the other BMAC individuals and to ancient Neolithic and Chalcolithic Turan groups, without the Steppe component (red) that is found in Iron Age individuals from Turan and outliers from BMAC (Figure 2; Supplementary Figure S3). We also estimated with f3-outgroup statistics of the form f3 (Mbuti; *ULG75*, PopX) that *ULG75* shares the most genetic drift with Turan groups from Chalcolithic to Late Bronze Age (Supplementary Figure S3). By comparing these results to those of the f3 (Mbuti; BMAC populations, PopX), we find that values observed for Ulug-depe are highly correlated with those obtained for other BMAC individuals (r2 = 0.93, *p* = 0) (Supplementary Figure S4).

**FIGURE 3:**
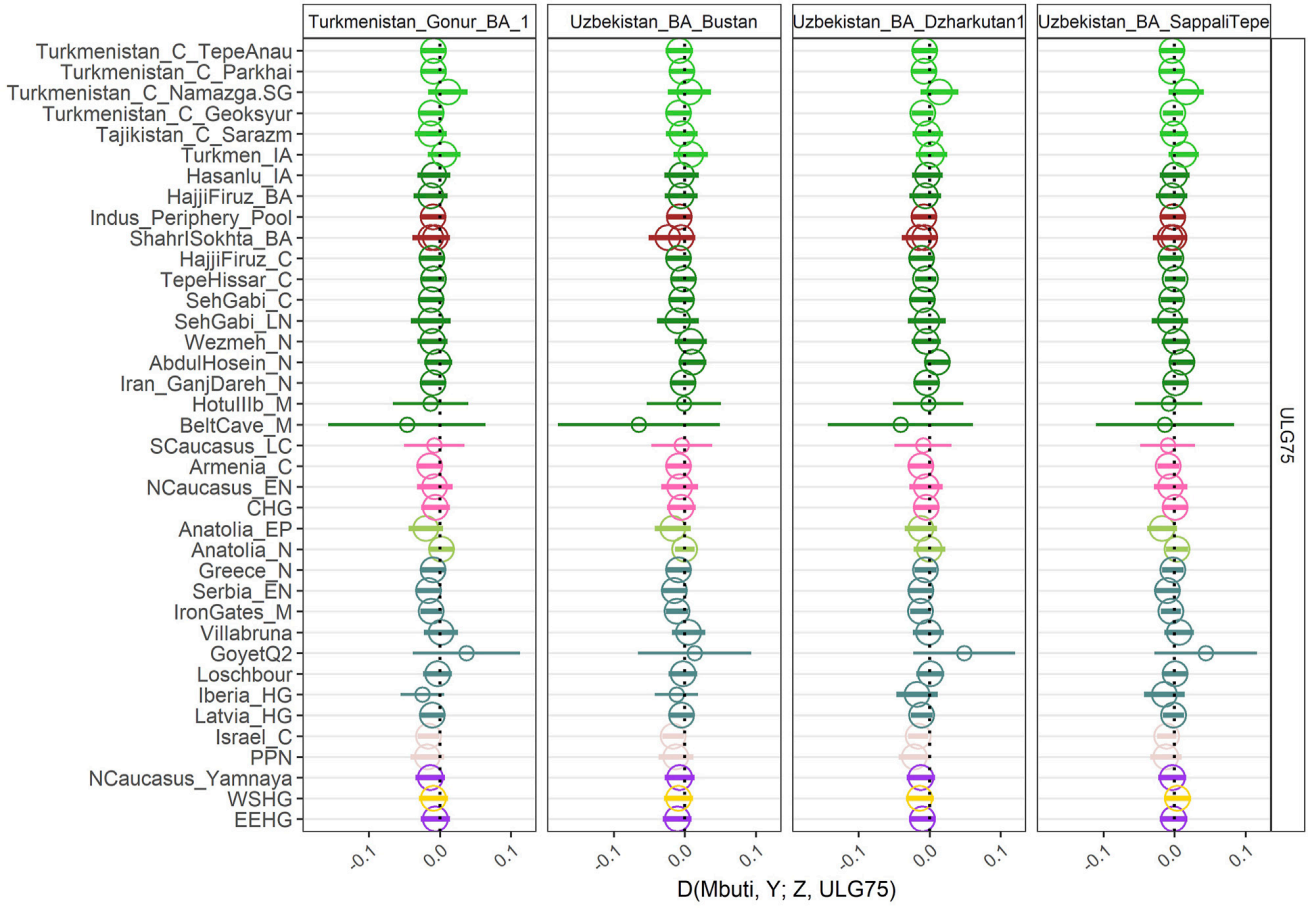
Genetic homogeneity of *ULG75* with other BMAC populations. D-statistics of the form D (Mbuti, Ancient Pop; BMAC, *ULG75*). The hollow point indicates a null D-statistic (|Z|<3). A small point indicates that the D-statistic was calculated with less than 50,000 SNPs.

To confirm that our individual forms a homogeneous group with the already published BMAC individuals, we also calculated D-statistics D (Mbuti, Ancient population; *ULG75*, BMAC). For all the D-statistics, we only obtained null D-stats (Figure 3) further reinforcing that *ULG75* belongs to the BMAC cluster. To fully test the cladality of *ULG75* with BMAC regarding eastern outlier from the Bronze Age, we performed qpAdm analysis with only previously published BMAC individuals as a unique source for *ULG75* and outliers populations, such as Shahr I Sokhta, the Indus Periphery Pool cluster, and Chalcolithic individuals from Seh Gabi. We did obtain non-significant results for all the tests, meaning the clade formed by *ULG75* with BMAC could not be broken.

Eventually, we tried to model *ULG75* as the BMAC groups (formed by several individuals from different sites) were modeled in Narasimhan et al. (2019). All the models worked, but with notably bigger standard error for the admixture percentage estimation, probably because we only considered one individual of limited coverage. We were able to confidently model *ULG75* (*p*-values = 0.46 and 0.63) as the product of an admixture of 54–63% (+/−10%) of the Chalcolithic population from Geoksyur, 12% (+/−5%) of Chalcolithic population from Hajji Firuz or 19% (+/−9%) from Seh Gabi, both in Iran, and 24% (+/−7%) of populations from the Indus Periphery. For the rest of the models, we obtained *p*-values above 0.05, but contributions from the admixed populations differed from what Narasimhan et al. (2019) obtained. Mostly, *ULG75* seems to be better modeled with only one contribution from the Bronze Age population, genetically part of the BMAC cluster, of Shahr-i-Shokhta (Iran), as nested models—inferring *ULG75* as 100% as Shahr-i-Shokhta—in our 3-way or 2-way admixtures could not be rejected (*p*-value > 0.05).

### Genetic continuity between BMAC group and modern Indo-Iranian populations

By computing D-statistics D (Mbuti, A selection of Ancient Populations (Y); Indo-Iranians, *ULG75*), we observed genetic continuity between *ULG75* and modern Indo-Iranian (Supplementary Figure S5) with clear gene flows from different steppe populations in Tajiks. We first obtained negative D-statistics (Z < −3) for Tajiks and Alakul, Saka, and Eastern European hunter-gatherers (from the Pontic Steppe), which points to a gene flow originating from the western or central steppes. We also have negative D-statistics of the form D (Mbuti, XiongNu/Shamanka, Tajiks, *ULG75*), which indicates a gene flow from the Eastern steppe with a strong Baikal ancestry.

We do not obtain significantly negative D-stats for Yagnobis, but the closer genetic proximity between *ULG75* and Iranian farmers from Ganj Dareh and Chalcolithic populations from Turan than Yagnobis and Tajiks [positive D (Mbuti, Iran_N/Turan_C; *ULG75*, Tajiks/Yagnobis)] indicates that the latter lost Iranian Neolithic ancestry since Bronze Age, probably linked to a gene flow from a population with low Iranian Neolithic ancestry, like most of the Steppe populations. Nevertheless, the low coverage of *ULG75* may not give us enough sensitivity to identify all the gene flows.

## Discussion and conclusion

### Genetic homogeneity in bronze age BMAC

Ancient DNA analyses of a Middle Bronze Age individual from Ulug-depe, *ULG75*, show that it belongs to the genetic BMAC cluster, represented by individuals from Gonur-depe, Dzharkutan, Bustan, and Sappali-tepe. This reinforces the integration of Ulug-depe into the Oxus Civilization, at a population level. Strontium and oxygen isotope analyses of Early and Middle Bronze Age individuals from Ulug-depe (Kroll et al., n.d.) have shown remarkable mobility in earlier periods, which clearly decreased with the rise of the BMAC. This inter-site mobility, found by the isotopic results, during the beginning of the Oxus Civilization has certainly contributed to the observed genetic homogeneity between the BMAC settlements.

On the other hand, previous genetic analyses on Dzharkutan, Gonur-Depe, Bustan, and Sappali-tepe Bronze Age populations have shown that in addition to the BMAC genetic cluster, first- or second-generation migrants from various parts of Eurasia were present within the Oxus. Interestingly, these outliers cluster with two different populations: one is related to steppe populations, from Northern Central Asia, while the second to the population from the Indus periphery. Due to the poor preservation of DNA in Ulug-depe, a comparison of the genetic diversity inside the site was not feasible. However, the absence of genetic ancestry coming from either of these populations in *ULG75* suggests if these migrants were present in Ulug-depe, they were not part of the ancestors of this individual. From an isotope point of view, the individuals contemporaneous of *ULG75* revealed little mobility and extensive use of the direct surrounding; no foreigners could have been identified (Kroll et al., n.d.).

### Origin and legacy of bronze age BMAC

As in Narasimhan et al. (2019), we observed that the BMAC individuals are largely derived from local, Chalcolithic populations similar to those found in Geoksyur. This genetic continuity matches with the long-term occupancy of several BMAC settlements and particularly Ulug-depe.

From a genetic point of view, the non-outlier BMAC individuals are strongly related to southerner populations such as the Iranian Neolithic, an observation that is also congruent with archaeological observations. The Turan archaeological sequence appears to mirror those found in South-western Asia. As early as the late seventh millennium BC, the Neolithic communities of South Central Asia have the same pattern of subsistence as those in the Near East and Iran; by the end of the fourth millennium, they also share the technological base for the production of pottery, metal, irrigation, etc., with numerous shreds of evidence showing material contact with the Iranian Plateau (Dani and Masson, 1992). These contacts may have implied gene flow as well and participate in the genetic homogeneity in this vast area.

This genetic continuity still goes on. A comparison of modern Indo-Iranian populations with the ancient genome (Guarino-Vignon et al., 2022) has shown genetic continuity with the BMAC cluster, with a high admixture with steppe populations, that occurred at the end of the BMAC. The first genetic outliers observed in several BMAC individuals may represent the very beginning of this demographic event. We also find evidence of gene flow from the East after the Iron Age, best modeled by XiongNu populations. This result is consistent with what has been found previously in Guarino-Vignon et al. (2022), reinforcing the XiongNu as a good proxy for modeling the gene flow that formed the Turko-Mongol populations and admixed with the local Indo-Iranian populations.

A more limited gene flow with the South-Asian populations has also been evidenced in Tajiks but not in Yagnobis, suggesting that it occurred mainly after the split between these two Indo-Iranian–speaking groups. On the other hand, outliers with a high amount of the Indus Periphery group are found in several BMAC groups, such as Bustan, and the *ULG75* genome can be modeled with as much as 24% of its ancestry derived from Indus Periphery. Thus, the small amount of South-Asian ancestry found in the modern Turan population may be explained by an ancient, low, continuous, gene-flow.

This analysis shows the strength of adding ancient DNA data to better understand the evolution of genetic diversity in Central Asia. Several questions remain about the timing of the different demographic events and call for more paleogenetic data, despite the difficulties due to the low preservation of ancient DNA in this warm, arid region.

## Data Availability

The datasets presented in this study can be found in online repositories. Study accession number: ERP135732 or PRJEB51133.
