# Supplementary material for: Genetic analysis of a bronze age individual from Ulug-depe (Turkmenistan)
Source: Front Genet. 2022 Aug 22;13:884612. doi: 10.3389/fgene.2022.884612 (PMC9441711; doi:10.3389/fgene.2022.884612)
Supplement: Supplementary file 4 [file Table1.DOCX]

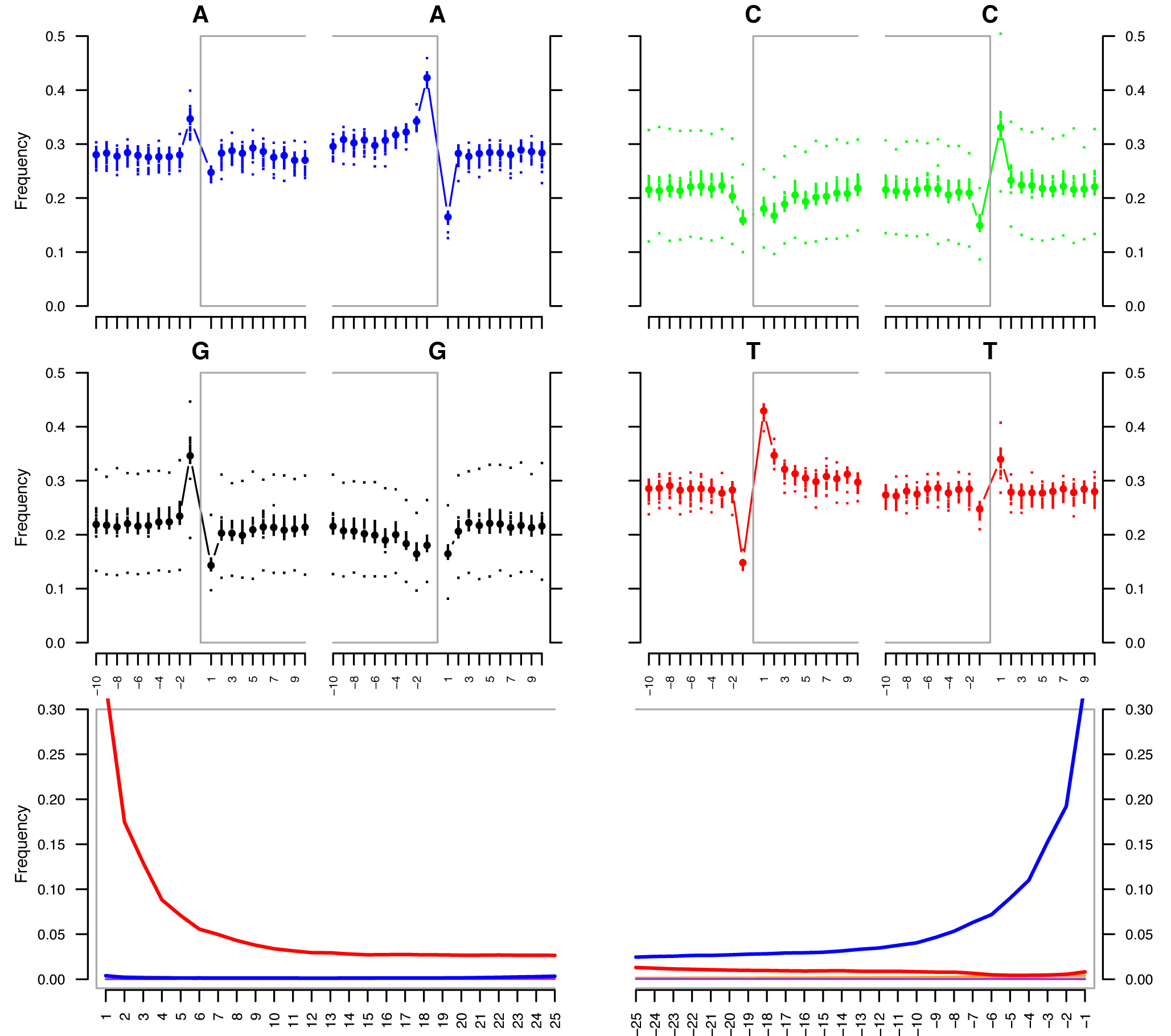


**Supplementary Figure 1.** Fragment incorporation plot obtained from human reads in ULG75, obtained through MapDamage 2. The profile shows damage patterns characteristic of authentic ancient DNA: the DNA fragmentation pattern shows an excess of purines at the genome position preceding the read start; and an increase of C -> T (G -> A) at the 5’ end (3’ end) display in blue (red).

**Supplementary Figure 2.** Full ADMIXTURE analysis for all modern (A) and ancient (B) individuals (Figure done with ggplot2 v. 3.3.3 R package https://cran.r-project.org/web/packages/ggplot2/index.html).


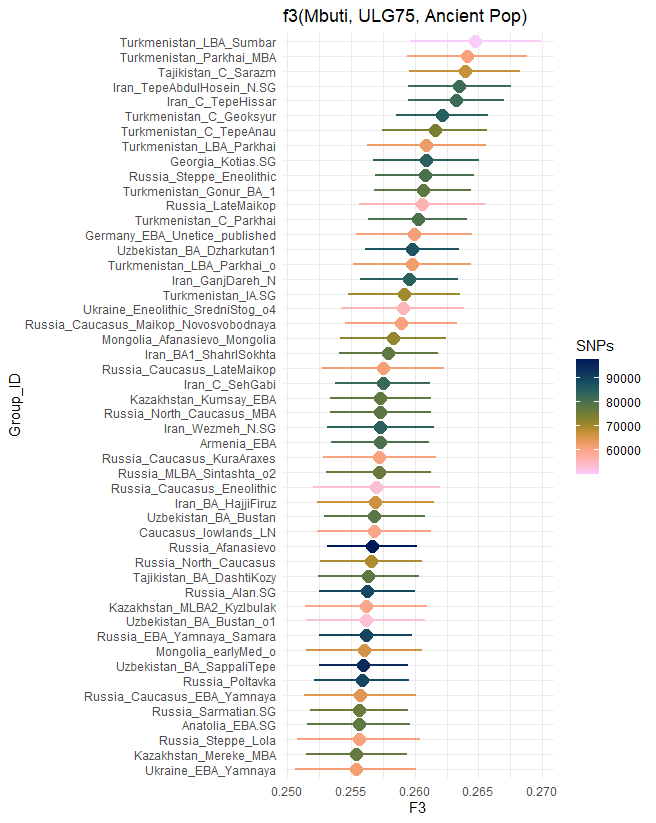


**Supplementary Figure 3.** 50 best f3-outgroup statistics of the form f3(Mbuti, *ULG75*, Western Eurasian populations), calculated with more than 50,000 SNPs. Name of genetic groups derive from 1240k dataset. Colors indicate the number of SNP retained in the analysis.


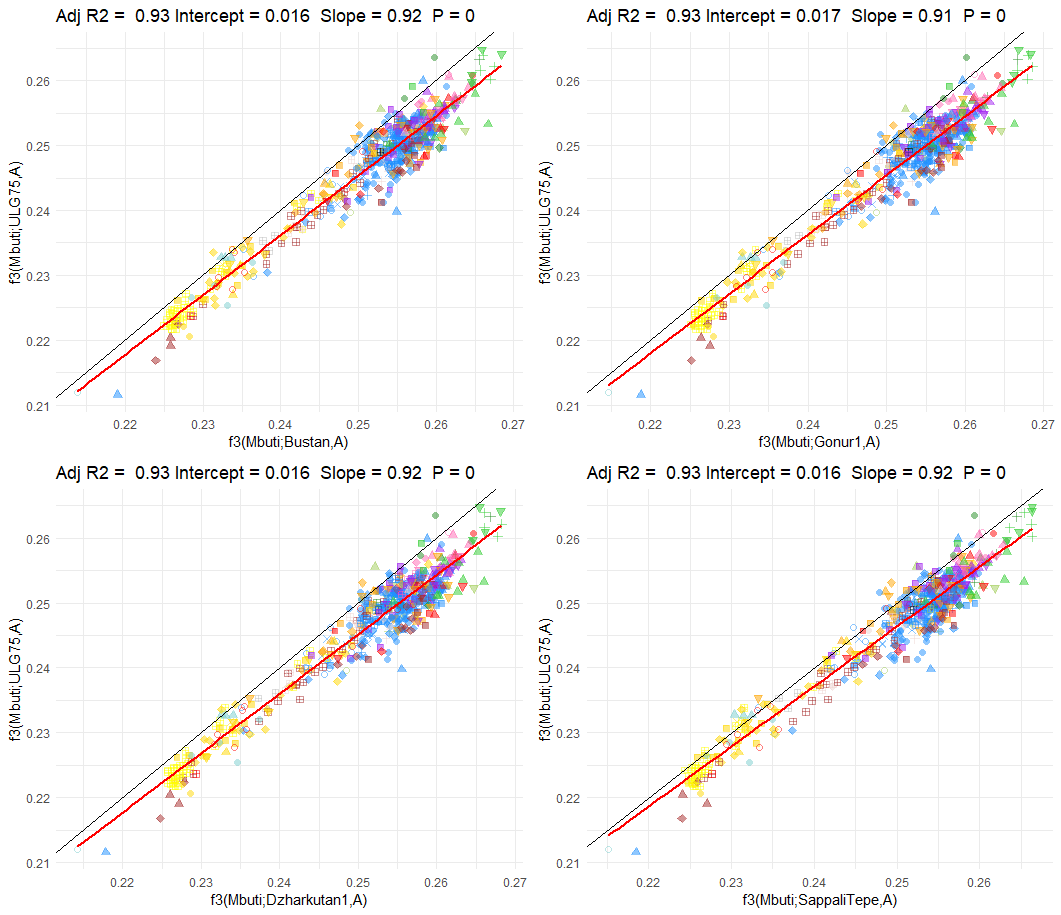


**Supplementary Figure 4.** Correlation between the f3-outgroup values (calculated with more than 50,000 SNPs) of the form f3(Mbuti, ULG75, Western Eurasian populations) and f3(Mbuti, BMAC, Western Eurasian populations) were BMAC groups are non outliers individuals from Bustan, Dzarkutan, Sappalli tepe and Gonur. Color code of Western Eurasian populations is the same as in Figure 2A.

**Supplementary Figure 5.** D-statistics D(Mbuti, Y; Indo-Iranians, ULG75) were Y is various proto-historic Eurasian populations. Tajiks and Yagnobi populations represent Indo-Iranians.

**Supplementary Table 1.** Contextual information for published ancient genome-wide data.

**Supplementary Table 2.** Contextual information for published modern genomes from all Eurasia.

**Supplementary Table 3.** Contextual information for modern genome from Central Asia (Shotgun).

**Supplementary Table 4.** All D-stat of the form D(Mbuti, Ancient pop;BMAC populations, ULG75), significantly positive or negtaive D-stat are highlighted in green

**Supplementary Table 5.** All D-stat of the form D(Mbuti, Ancient pop; TJE/TJY, ULG75), significantly positive or negative D-stat are highlighted in green.

**Supplementary Table 6.** All f3-outgroup of the form f3(ULG75 or BMAC population, Ancient pop; Outgroup)

**Supplementary Table 7.** Replicated qpAdm models from Narasimhan et al. 2019 for ULG75
