# Supplementary material for: Genetic analysis of a bronze age individual from Ulug-depe (Turkmenistan)
Source: Front Genet. 2022 Aug 22;13:884612. doi: 10.3389/fgene.2022.884612 (PMC9441711; doi:10.3389/fgene.2022.884612)

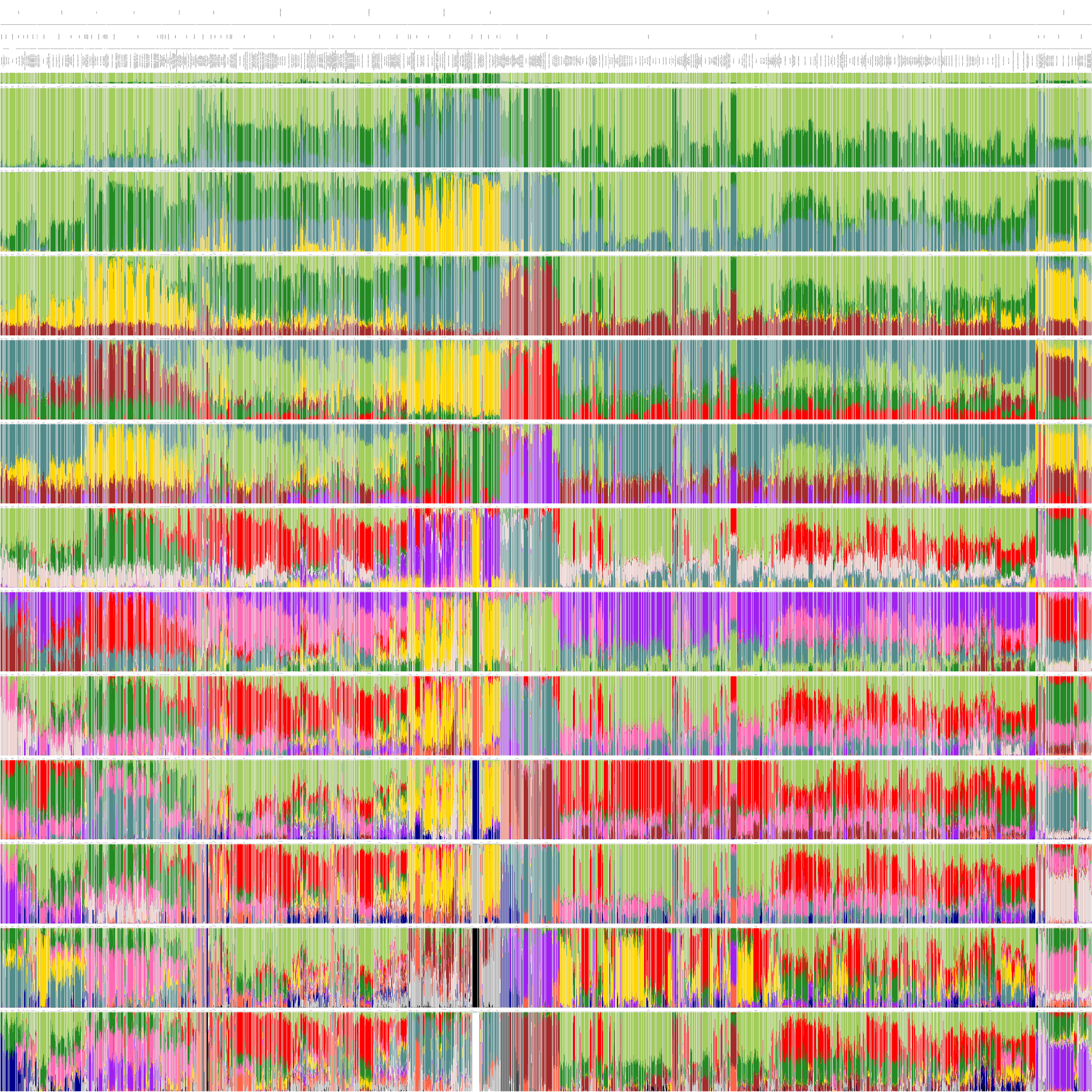

Supplement: Supplementary file 1 [file DataSheet2.PDF]
