# Supplementary material for: Genetic analysis of a bronze age individual from Ulug-depe (Turkmenistan)
Source: Front Genet. 2022 Aug 22;13:884612. doi: 10.3389/fgene.2022.884612 (PMC9441711; doi:10.3389/fgene.2022.884612)
Supplement: Supplementary file 6 [file DataSheet1.PDF]

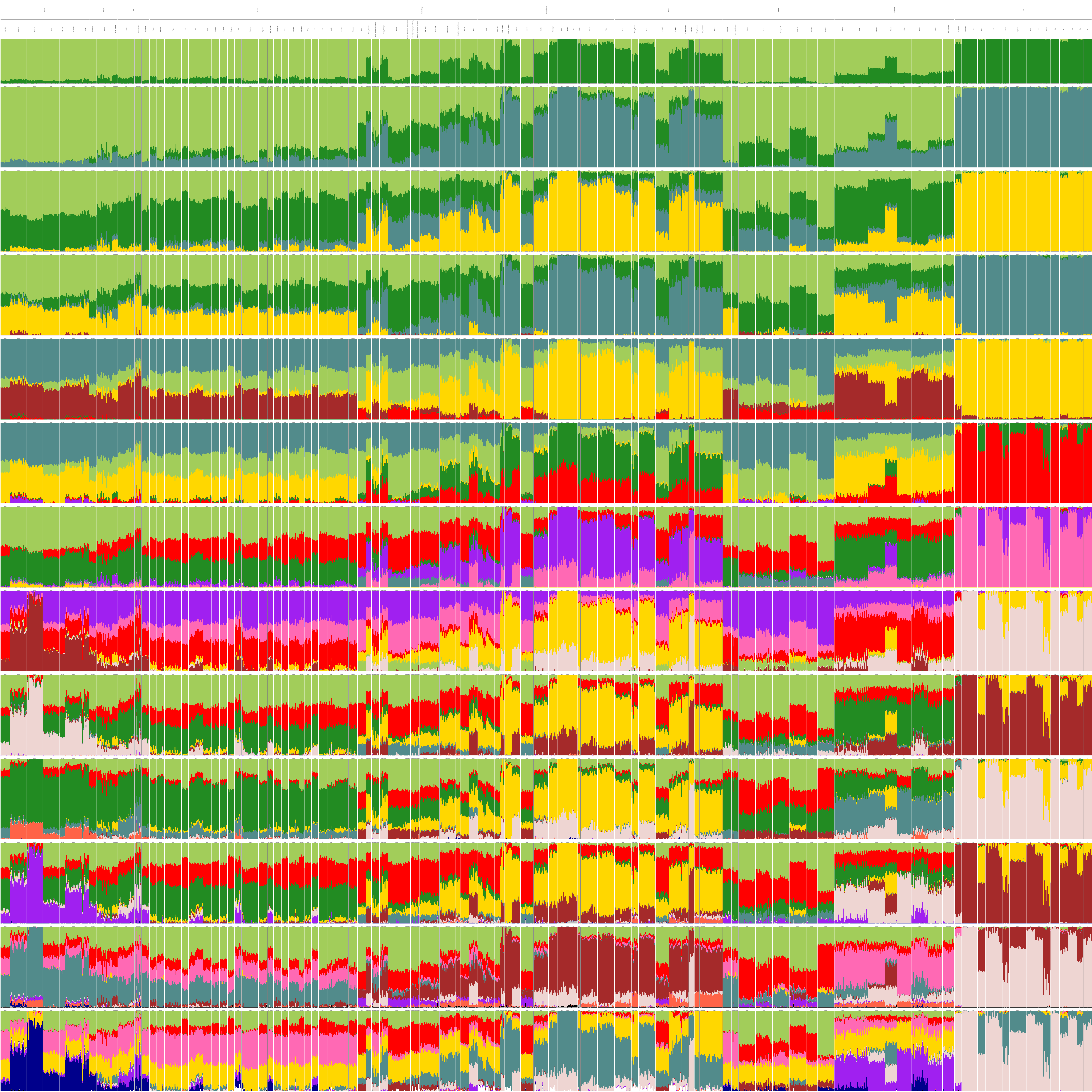
Levant
Anatolia
Iran
Caucasus
CentralSteppe
EasternSteppe
Baikal
Europe
SouthAsia
NA
Assyrian
BedouinA
BedouinB
Druze
Jew_Iraqi
Palestinian
Syrian
Jew_Turkish
Turkish
Turkish_Balikesir
Iranian
Iranian_Bandari
Jew_Iranian
Abazin
Abkhasian
Adygei
Avar
Azeri
Balkar
Chechen
Circassian
Darginian
Ezid
Georgian
Ingushian
Jew_Georgian
Kabardinian
Kaitag
Karachai
Kubachinian
Kumyk
Kurd
Lak
Lezgin
Ossetian
Tabasaran
Veps
Nogai_Astrakhan
Nogai_Karachay_Cherkessia
Nogai_Stavropol
Russian
Russian_Archangelsk_Krasnoborsky
Russian_Archangelsk_Leshukonsky
Russian_Archangelsk_Pinezhsky
Tatar_Kazan
Tatar_Mishar
Tatar_Siberian
Tatar_Siberian_Zabolotniye
Udmurt
Uyghur
Bashkir
Besermyan
Evenk_FarEast
Evenk_Transbaikal
Kalmyk
Karelian
Kazakh
Khamnegan
Nanai
Negidal
Nivh
Todzin
Tuvinian
Yakut
Altaian
Altaian_Chelkan
Buryat
Chuvash
Khakass
Khakass_Kachin
Mongola
Shor_Khakassia
Shor_Mountain
Tubalar
Armenian
Armenian_Hemsheni
Basque
French
Italian_North
Mordovian
Orcadian
Sardinian
Balochi
Brahui
Burusho
Hazara
Kalash
Makrani
Pathan
Sindhi_Pakistan
Cambodian
China_Lahu
Dai
Daur
Han
Hezhen
Japanese
Miao
Naxi
Oroqen
She
Tu
Tujia
Xibo
Yi
